# Mathematical analysis in the design of digital artery-based V–Y advancement flap in treating proximal interphalangeal joint flexion contracture

**DOI:** 10.1186/s12891-023-06158-7

**Published:** 2023-01-17

**Authors:** Po-Han Su, Cheng-En Hsu, Tsung-Yu Ho, Bor-Han Wei, Wei-Chih Wang, Yung-Cheng Chiu

**Affiliations:** 1 School of Medicine, China Medical University, Taichung City, 404 Taiwan; 2 Department of Orthopedic Surgery, China Medical University Hospital, China Medical University, No. 2, Xueshi Rd, Taichung City, 404 Taiwan; 3 Sports Recreation and Health Management Continuing Studies-Bachelor’s Degree Completion Program, Tunghai University, Taichung, 407 Taiwan; 4 Department of Orthopedic Surgery, Taichung Veterans General Hospital, Taichung, 407, Taiwan; 5 Department of Orthopedic, Chung-Kang Branch, Cheng-Ching General Hospital, Taichung, Taiwan

**Keywords:** Proximal interphalangeal joint, Distal interphalangeal joint, Middle phalanx, V–Y advancement flap, Proximal phalanx, Flexion contracture

## Abstract

**Background:**

The digital artery-based V–Y advancement flap is a widely used flap for soft tissue coverage in the treatment of flexion contracture of the proximal interphalangeal (PIP) joint. A standard method for the flap design and a mathematical method to predict the advance distance have not been well established. In this study, we proposed a simplified method for the design of V–Y advancement flaps based on digital arteries and used a geometric model to predict the advance distance for the flexion contracture correction surgery.

**Methods:**

According to the general concept of hand flap design and law of cosine, we proposed three principles in the design of the digital artery-based V–Y advancement flap that should be followed. Since 2021 to 2022, finger geometric data of 120 fingers (index, middle, ring, and small fingers) from 30 healthy participants were collected and analysed to evaluate the necessary advance distance and flap tip angle for PIP flexion contracture correction of different fingers by our flap design method.

**Results:**

The middle finger needed a significantly longer advance distance compared to other fingers in the same degree flexion contracture correction. The ring finger had the largest length-to width ratio and smallest flap tip angle among the four fingers in the V–Y flap design. No vertical scar crossed the flexion creases and flap tip angle < 20° was found in the tentative V–Y flap design for the 120 fingers.

**Conclusions:**

Our flap design method provides a proper advance distance and flap length-to-width ratio without common skin complications in the flap design for PIP flexion contracture of index, middle, ring and small fingers. This geometric model provides a mathematical basis for prediction of advance distance and flap tip angle in the design of a digital artery-based V–Y advancement flap.

**Supplementary Information:**

The online version contains supplementary material available at 10.1186/s12891-023-06158-7.

## Background

Flexion contracture of the proximal interphalangeal (PIP) joint is mainly caused by trauma and can impair the function of the entire hand; ensuing adverse effects may jeopardise a patient’s daily activity. Common reasons for PIP joint flexion contracture include soft tissue fibrosis, tendon adhesions, and joint capsular contracture [1, 2]. When functional range of motion (ROM) cannot be obtained by conservative treatment, surgical treatment is indicated to facilitate improvement of flexion/extension arc of motion into a more functional range [3]. In severe cases, restoration of full PIP joint extension often creates a soft tissue defect on the volar aspect of the finger. Various flaps and skin grafting techniques have been developed to treat PIP joint contracture; however, shortage of skin and soft tissue defects after surgical release prevents the achievement of full extension of the PIP joint and early rehabilitation protocol. The digital artery-based V–Y advancement flap to treat PIP joint flexion contracture was first introduced by Kinoshita et al. [4]. Instead of a purely palmar incision, this flap is raised by making mid-lateral skin incisions on both sides of the finger and a volar V-shaped skin incision in the distal palm [5]. Freeing the digital neurovascular bundles from the surrounding tissue, this flap permits adequate distal advancement without the need for a skin graft for wound closure and offers reliable flap circulation. Although sufficient skin coverage may be expected, geometric analysis for predicting the advance distance of the flap and the correctable contracture angle has not been developed. In this study, we proposed a mathematical method for estimating the advance distance of the flap for PIP joint flexion contracture. We also developed a simplified method for the design of V–Y advancement flaps and predicted the advance distance in each finger when a 90° PIP joint flexion contracture is corrected according to the finger geometric data of 120 fingers from 30 healthy participants.

## Methods

### Geometric model

According to the general concept of hand flap design, three principles in the design of the digital artery-based V–Y advancement flap should be followed:

1. During flap elevation, the tip of the V–Y flap should not be proximal to the distal palmar crease; after distal flap advancement, the tip of the V–Y flap should not exceed the palmar digital crease to avoid scar contracture that may compromise metacarpophalangeal (MCP) joint motion.
2. The distance of the V–Y flap advancement should not exceed 14 mm to avoid arterial insufficiency and nerve damage [6].
3. The length-to-width ratio of any flap should not exceed 2:1 to prevent blood circulation disorders or necrosis at the distal end of the flap [7]

**Fig. 1:**
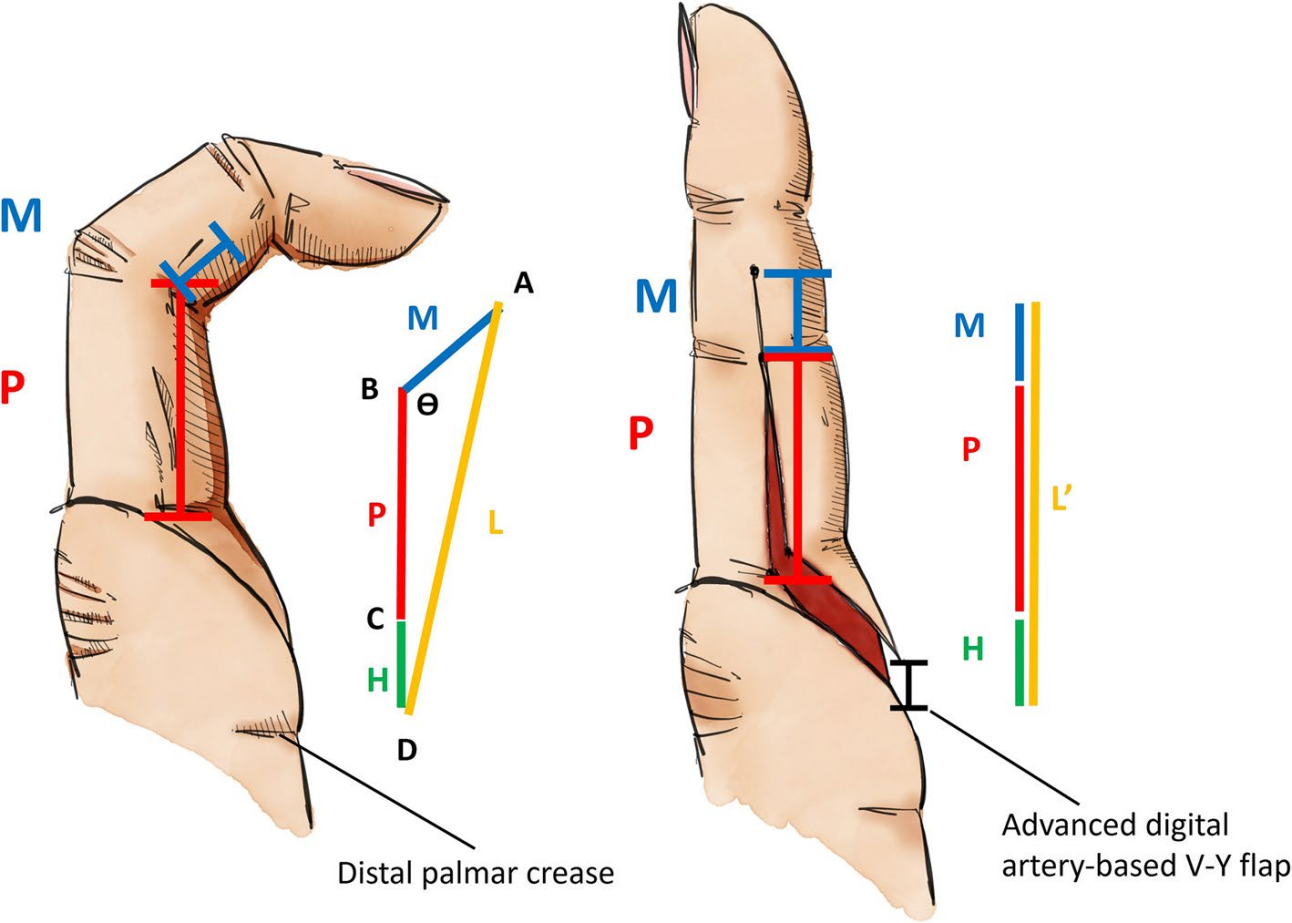
The length of the skin defect and that of advance distance of the V–Y flap should be L'-L. The distance from the midpoint of the middle phalanx to its base on the contralateral hand is labelled as L'. The same interval on the affected finger is named L ($\overline{AD }$; orange line). Line P ($\overline{BC }$; red line) is the length of the proximal phalanx; line M ($\overline{AB }$; blue line), half the length of the middle phalanx; and line H ($\overline{CD }$; green line), the height of the flap, which also represents the distance of the advancement of the V–Y flap. The angle θ (∠ABC) indicates the angle of limitation of extension in the PIP joint with flexion contracture

**Fig. 2:**
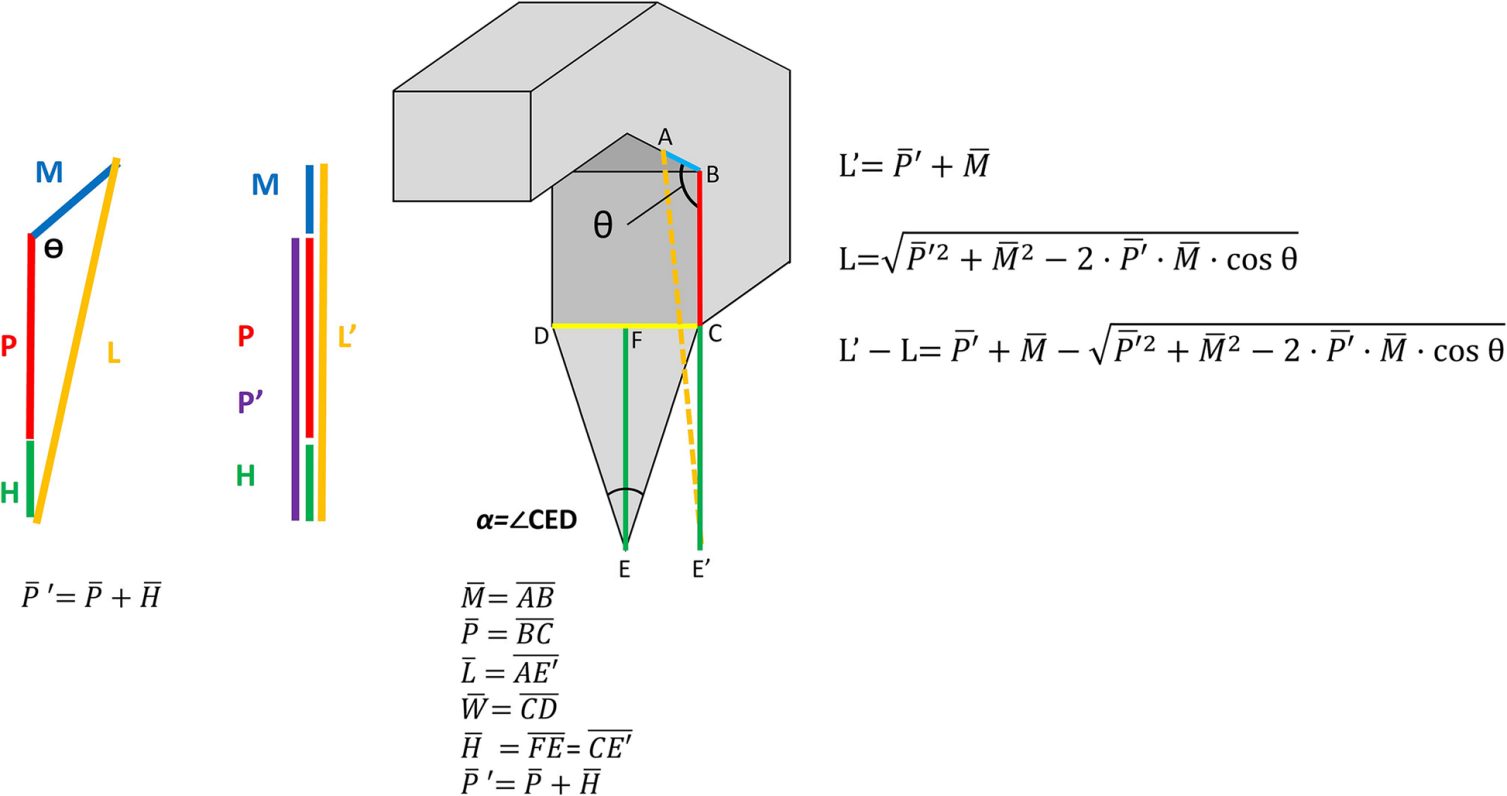
Illustration of the model of a contracted digit. For simplifying the calculation, make line P'’ = P + H (the combined length of line P and line H; purple line). Triangle CDE is the area of the digital artery-based V–Y advancement flap. The distance from the midpoint of the middle phalanx to its base on the contralateral hand is labelled as L'. The same interval on the affected injured finger is named L ($\overline{AC }$; orange dashed line). Line P ($\overline{BC }$; red line), length of proximal phalanx; line M ($\overline{AB }$; blue line), half of length of middle phalanx; and line W ($\overline{CD }$; yellow line), width of digital base. When Line H ($\overline{FE }$; brown line) = 1.5 W, α (angle of flap apex) = ∠CED = 36.9°. Angle θ (angle of limitation of extension in the PIP joint with flexion contracture) = ∠ABC

We hypothesised that the correlation between the advance distance of the V–Y advancement flap and the correctable PIP joint angle can be accurately estimated according to certain quantitative parameters demonstrated in Fig. 1: 1) on the contralateral uninjured finger, the length from the midpoint between the distal interphalangeal (DIP) and PIP creases to the palmar digital crease, was measured as L' and 2) the same interval was measured on the injured digit (L). Based on our model, to achieve full extension of the PIP joint, the distance of advancement of our V–Y advancement flap should be L'-L. In Fig. 2, Line P ($\overline{\mathrm{BC} }$) is the length of the proximal phalanx of the injured finger. Line M ($\overline{\mathrm{AB} }$) is half the length of the middle phalanx. Line W ($\overline{\mathrm{CD} }$) is the width of the digital base. Line H ($\overline{\mathrm{FE} }$) represents the height of the flap (The length of Line H varies between the interval of distal palmar crease and palmar digital crease. The length-to-width ratio of any flap should not exceed 2:1, and the tip of the V–Y flap should not be proximal to the distal palmar crease as well). To simplify the calculation, we make line P' = P + H (the combined length of line P and line H; purple line). Angle α (∠CED) is the angle of the flap apex. The flap is an isosceles triangle. The angle θ (∠ABC) is defined as the angle of extension limitation in the PIP joint with flexion contracture. The distance of V–Y flap advancement (L'-L) can be calculated using the law of cosines of triangles:

The advance distance of V–Y flap L' $$-$$ L = $$\overline{P}^{\prime}$$+ $$\overline{M }$$$$- \sqrt{{\overline{P}}^{{\prime}{2}}+{\overline{M} }^{2}-2\cdot{\overline{P}}^{\prime}\cdot{\overline{M}}\cdot\text{cos}\;{\uptheta}}$$

To calculate the flap advance distance needed to correct a 90°-flexion contracture. ${\uptheta}$=90° i.e., $\mathrm{cos}\;{\uptheta}$=0 is substituted into the equation:

$$\mathrm L'-\mathrm L={\overline{P}}^{\prime}+\overline{M }- \sqrt{{\overline{P}}^{{\prime}{2}}+{\overline{M} }^{2}}$$

To estimate the flap advance distance, hand geometric data of 120 fingers from 30 adults (15 males and 15 females) were collected. The length and width of the fingers were recorded and analysed. The study protocol was approved by the Research Ethics Committee of the China Medical University Hospital, Taichung, Taiwan (protocol ID: CMUH110-REC1-166) and conducted in accordance with the ethical principles of the Helsinki Declaration. The inclusion criteria were healthy adults over 20 years of age. The exclusion criteria were people younger than 20 years of age, those who had digital contracture or dysfunction, and those with open wounds or scars on their hands.

**Fig. 3:**
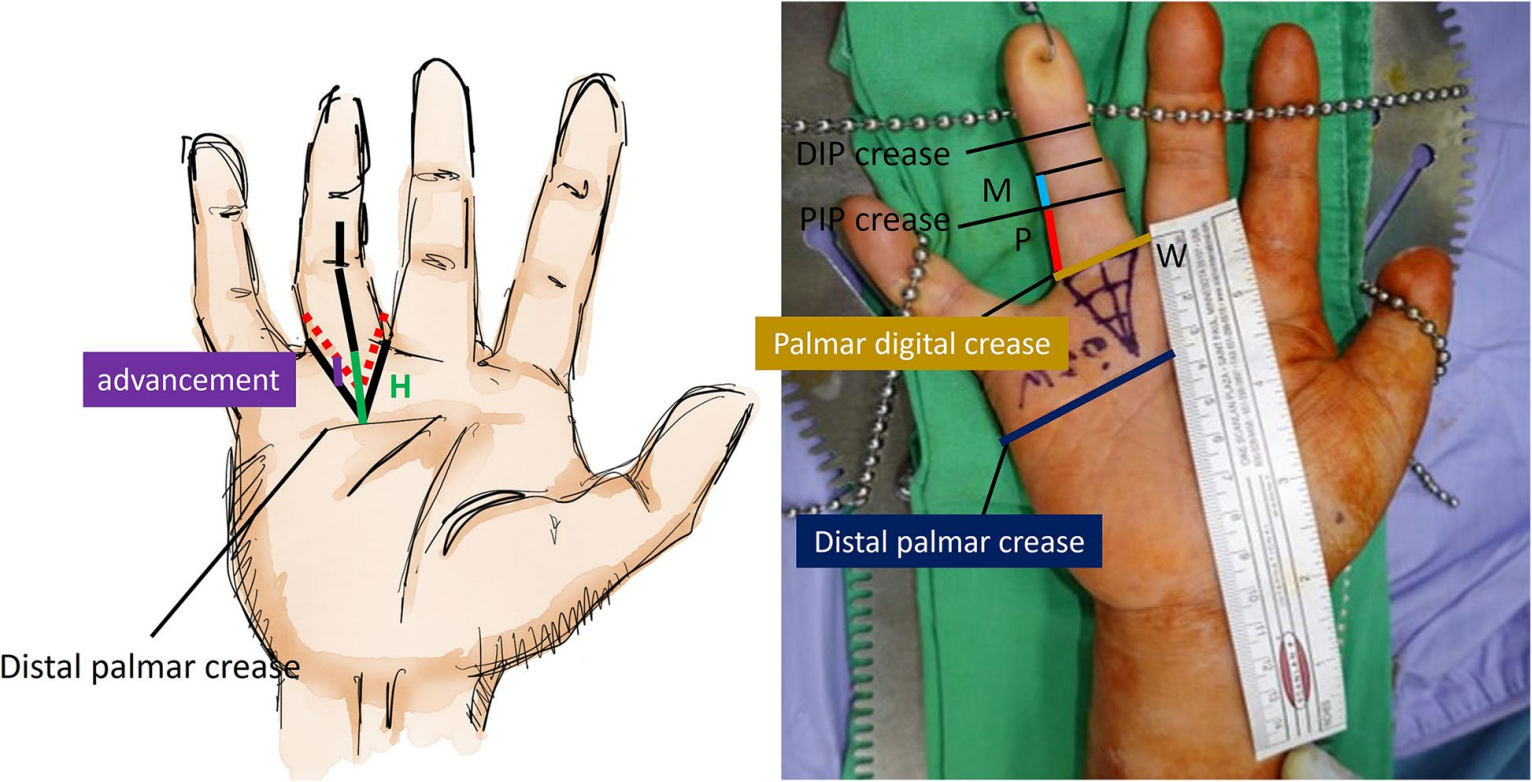
Left: The illustration of the advancement of the V–Y flap (purple line; the distance of advancement does not exceed the height of the flap). Right: width of the digital base (W; yellow line) was defined as the distance from the radial to the ulnar sides of the digit over the palmar digital crease; the length of the proximal phalanx (P; red line) was defined as the distance between the PIP and palmar digital creases; and half the length of the middle phalanx (M; blue line) was defined as half the distance between the DIP and PIP creases

Measurement of the length of the proximal phalanx (P), half the length of the middle phalanx (M), the length from the palmar digital crease to the distal palmar crease (H), and the width of the digital base (W) were measured by an orthopaedic surgeon with five years of surgical experience. To avoid confusion with multiple parallel creases in palm, we suggest setting the standard line with palm slightly bended, which highlights the crease. The details of this measurement method are shown in Fig. 3. W was defined as the distance from the radial to the ulnar sides of the digit over the distal palmar crease; the length of P was defined as the distance between the PIP and distal palmar creases; and half the length of M was defined as half of the distance between the DIP and PIP creases.

### Surgical procedure

For all patients with post-traumatic flexion contracture of the PIP joint, the severity of joint contracture was determined according to the intraoperative findings, and optimal treatment strategies were customised for individual patients. To achieve this goal, wide-awake local anaesthesia without tourniquet (WALANT technique) [8] was adopted to offer the surgeon a high flexibility in tailoring the operative strategy according to the intraoperative feedback from the patient.

**Fig. 4:**
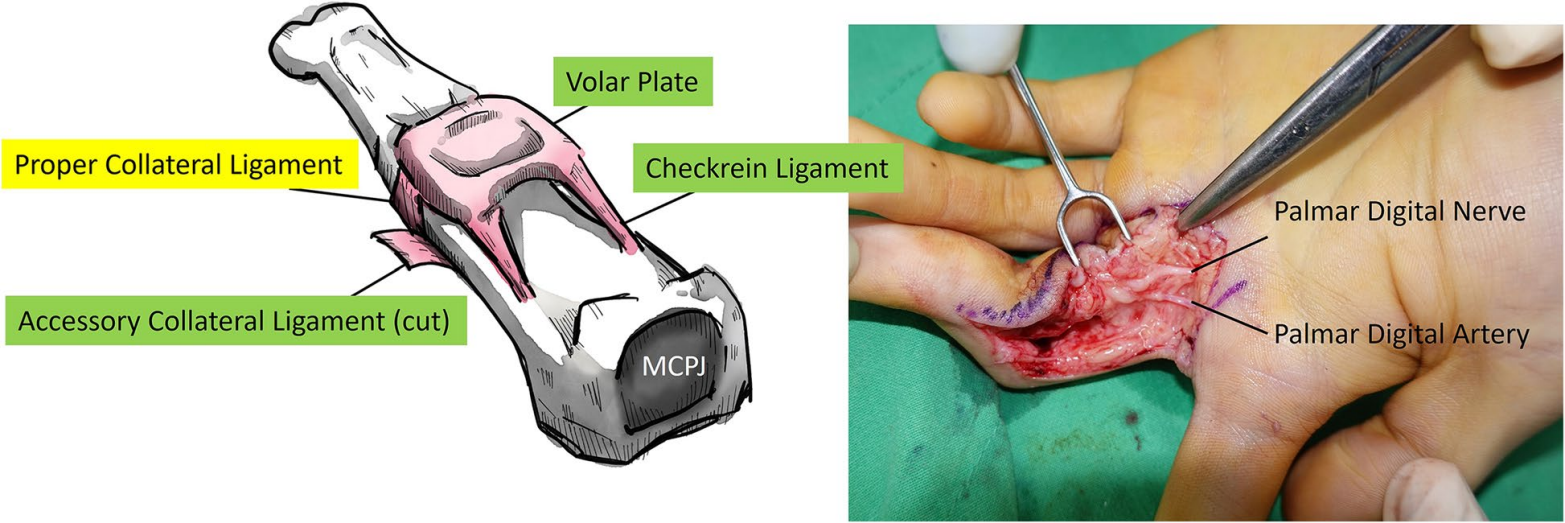
Illustration of the volar view of the PIP joint anatomy. The proper collateral ligament (yellow) is preserved intraoperatively to maintain joint stability. The accessory collateral ligaments, checkrein ligaments, and proximal margin of the volar plate (green) are released. MCPJ = metacarpophalangeal joint

Using the WALANT technique, WALANT solution (10 mL 1% lidocaine and 1:100,000 diluted epinephrine) was injected into the surgical site according to the suggested dose and location [9]. In a case of correcting 90°-flexion contracture of PIP joint, our treatment protocol for post-traumatic PIP joint flexion contracture was as follows: first, through bilateral mid-lateral incision, release of the accessory collateral ligaments, checkrein ligaments, and proximal margin of the volar plate, was performed (Fig. 4). The proper collateral ligament was preserved to maintain the essential stability of the PIP joint. If the adhesion of the flexor tendon or joint capsule were noted during the operation, tenolysis or capsulotomy were performed in the same step. After freeing up the thickened scar tissue around the volar plate, arthrolysis of the PIP joint was performed using a small dissector through the window of the proximal-margin-detached volar plate. Active range of motion (AROM) was tested with the patient under local anaesthesia. Once the improvement was deemed unsatisfactory, the second step, that is, FDS tendon insertion release, was performed. After releasing the FDS tendon insertion, the patient was asked to move their finger actively again to test the AROM of the PIP joint. If finger motion had still not returned to functional range, the third step, a V–Y advancement flap based on the digital artery, was performed.

A bilateral mid-lateral incision was made at the finger level and extended proximally to the palmer side in a V–Y fashion. The height (H) of the V–Y flap at the level of the palm was set according to our formula. The bilateral proper digital artery and nerve were included in the flap and dissected proximally until the level of bifurcation of the common digital artery to achieve good distal advancement of the flap. All the skin perforator vessels originating from the digital artery were carefully preserved to ensure perfusion of the skin flap. The V–Y advancement flap based on the digital artery was freed from the distal palm and moved distally. With the calculated advance distance according to our formula, most flexion contracted fingers are fully extended, and the resulting skin and soft tissue defects can be easily managed with a Y-shaped wound closure; the flap tip can still be nourished with sufficient blood supply.

### Statistical methods

Shapiro–Wilk test was performed to assess the normality of continuous variables. Student t test and ANOVA were used in the analysis of quantitative variables. Tukey's method was used for post-hoc analysis. Correlations were considered significant if p values were less than 0.05 (two-sided).

## Results

In total, 120 fingers from 30 healthy adults (15 males and 15 females) were collected and analysed. The average length of P and M based on sex, side, and fingers are summarized in Table 1.

**Table 1:** Comparison of the length of finger segment of 120 fingers in 30 adults

	M	*P*-value	P	*P*-value	H	*P*-value	W	*P*-value
All (n = 120)	11.0 ± 2.21		23.7 ± 4.21		24.1 ± 4.67		20.4 ± 2.94	
Gender		**0.041**		** < 0.001**		** < 0.001**		** < 0.001**
Male (n = 60)	11.6 ± 1.80		25.5 ± 3.74		25.6 ± 4.67		22.2 ± 2.43	
Female (n = 60)	10.3 ± 2.41		21.9 ± 3.89		22.6 ± 4.20		18.6 ± 2.22	
Finger		** < 0.05**		** < 0.05**		** < 0.05**		** < 0.05**
Index (n = 30)	11.0 ± 1.11	** < 0.05**^** M,S**^	24.7 ± 3.01	** < 0.05 **^**S**^	22.4 ± 3.37	** < 0.05**^**R**^	22.1 ± 2.61	** < 0.05**^** M,S**^
Middle (n = 30)	12.8 ± 1.61	** < 0.05**^**I.R,S**^	26.8 ± 3.19	** < 0.05**^**R,S**^	24.1 ± 5.16	** < 0.05**^**R**^	19.3 ± 2.66	** < 0.05**^**I,R,S**^
Ring (n = 30)	11.5 ± 1.31	** < 0.05**^** M,S**^	23.9 ± 3.62	** < 0.05**^** M,S**^	28.1 ± 3.48	** < 0.05**^**I,M,S**^	19.3 ± 2.49	** < 0.05**^** M,S**^
Small (n = 30)	8.5 ± 2.11	** < 0.05**^**I,M,R**^	19.4 ± 3.27	** < 0.05**^**I,M,R**^	21.9 ± 3.84	** < 0.05**^**R**^	21.0 ± 3.10	** < 0.05**^**I,M,R**^
Side		0.638		0.638		0.955		0.432
Right (n = 56)	11.1 ± 2.26		23.9 ± 4.19		24.1 ± 5.10		20.6 ± 2.81	
Left (n = 64)	10.8 ± 2.18		23.5 ± 4.25		24.1 ± 4.29		20.2 ± 3.05	

Values are reported as mean ± standard deviation (range). p values computed by student t test and *One-way ANOVA analysis

I: vs. index fingers

M: vs. middle fingers

R: vs. ring fingers

S: vs. small fingers

Significant longer M, P, H, and W were found in male participants than in female participants (P < 0.001). Significant difference was found in M, P, H, and W length among index, middle, ring, and small fingers (P < 0.001). No significant difference was found for M, P, H, and W length on different sides of hands.

The estimated advance distance for a 90°-PIP flexion contracture correction and the distance between palmar digital crease and distal palmar crease (H) for each finger by sex are shown in Table 2.

**Table 2:** Analysis of the advance distance for a 90° PIP flexion contracture correction and the distance between distal palmar crease and palmar digital crease (H) in 120 fingers of 30 adults

	Advance (mm)	*P*-value	H (mm)	*P*-value
Male				
Finger		< 0.05*		< 0.05*
Index (n = 15)	9.9 ± 0.53	** < 0.05 **^**S**^	23.1 ± 3.34	** < 0.05 **^**S**^
Middle (n = 15)	11.7 ± 1.00	** < 0.05 **^**I,S**^	26.0 ± 5.01	
Ring (n = 15)	10.8 ± 0.89	** < 0.05 **^**S**^	29.4 ± 3.33	** < 0.05 **^**I,S**^
Small (n = 15)	8.7 ± 1.43	** < 0.05 **^**I,M,R**^	23.9 ± 4.30	** < 0.05 **^**R**^
Female				
Finger		< 0.05*		< 0.05*
Index (n = 15)	9.5 ± 1.12	** < 0.05 **^**S**^	21.6 ± 3.27	** < 0.05 **^**R**^
Middle (n = 15)	10.7 ± 1.34	** < 0.05 **^**S**^	22.2 ± 4.68	** < 0.05 **^**R**^
Ring (n = 15)	9.8 ± 1.05	** < 0.05 **^**S**^	26.8 ± 3.21	** < 0.05 **^**I,M,S**^
Small (n = 15)	6.5 ± 1.29	** < 0.05 **^**I,M,R**^	19.9 ± 1.77	** < 0.05 **^**R**^

Values are reported as mean ± standard deviation (range). p values computed by student t test and *One-way ANOVA analysis

S: vs. small fingers

R: vs. ring fingers

M: vs. middle fingers

I: vs. index fingers

Significant less advance distance was found in the small finger than in the other three fingers (P < 0.05). Ring finger had the longest H, compared to that of the other three fingers. The advance distances in the index, middle, ring, and small fingers were less than the length of H.

The length–width ratio of tip and the angle of the V–Y flap (α) for each finger by sex are shown in Table 3.

**Table 3:** Analysis of flap tip angle in 120 fingers of 30 adults

	H/W	*P*-value	Flap tip angle (α)	*P*-value
Male				
Finger		< 0.05*		< 0.05*
Index (n = 15)	0.96 ± 0.125	** < 0.05 M**^**,R**^	55.4 ± 6.54	** < 0.05 M**^**,R**^
Middle (n = 15)	1.26 ± 0.232	** < 0.05 **^**I,S**^	44.6 ± 8.21	** < 0.05 **^**I,S**^
Ring (n = 15)	1.41 ± 0.173	** < 0.05 **^**I,S**^	39.6 ± 5.30	** < 0.05 **^**I,S**^
Small (n = 15)	1.17 ± 0.259	** < 0.05 M**^**,R**^	52.2 ± 7.52	** < 0.05 M**^**,R**^
Female				
Finger		< 0.05*		< 0.05*
Index (n = 15)	1.08 ± 0.166	** < 0.05 **^**R**^	50.5 ± 6.65	** < 0.05 **^**R**^
Middle (n = 15)	1.26 ± 0.311	** < 0.05 **^**R**^	45.1 ± 9.78	** < 0.05 **^**R**^
Ring (n = 15)	1.54 ± 0.209	** < 0.05 **^**I,M,S**^	36.6 ± 4.56	** < 0.05 **^**I,M,S**^
Small (n = 15)	1.23 ± 0.285	** < 0.05 **^**R**^	51.0 ± 6.06	** < 0.05 **^**R**^

Values reported as the mean ± one standard deviation (range). p values computed by student t test and *One-way ANOVA analysis

S: vs. small fingers

R: vs. ring fingers

M: vs. middle fingers

I: vs. index fingers

While significantly less V–Y flap tip angle was found for the ring finger compared to the other three fingers in the female participants, significantly less V–Y flap tip angle was noted for the ring and middle fingers compared to the other two fingers in the male participants (P < 0.05). All the V–Y flap tip angles were not > 60° in all fingers.

### Case examples

#### Patient 1

**Fig. 5:**
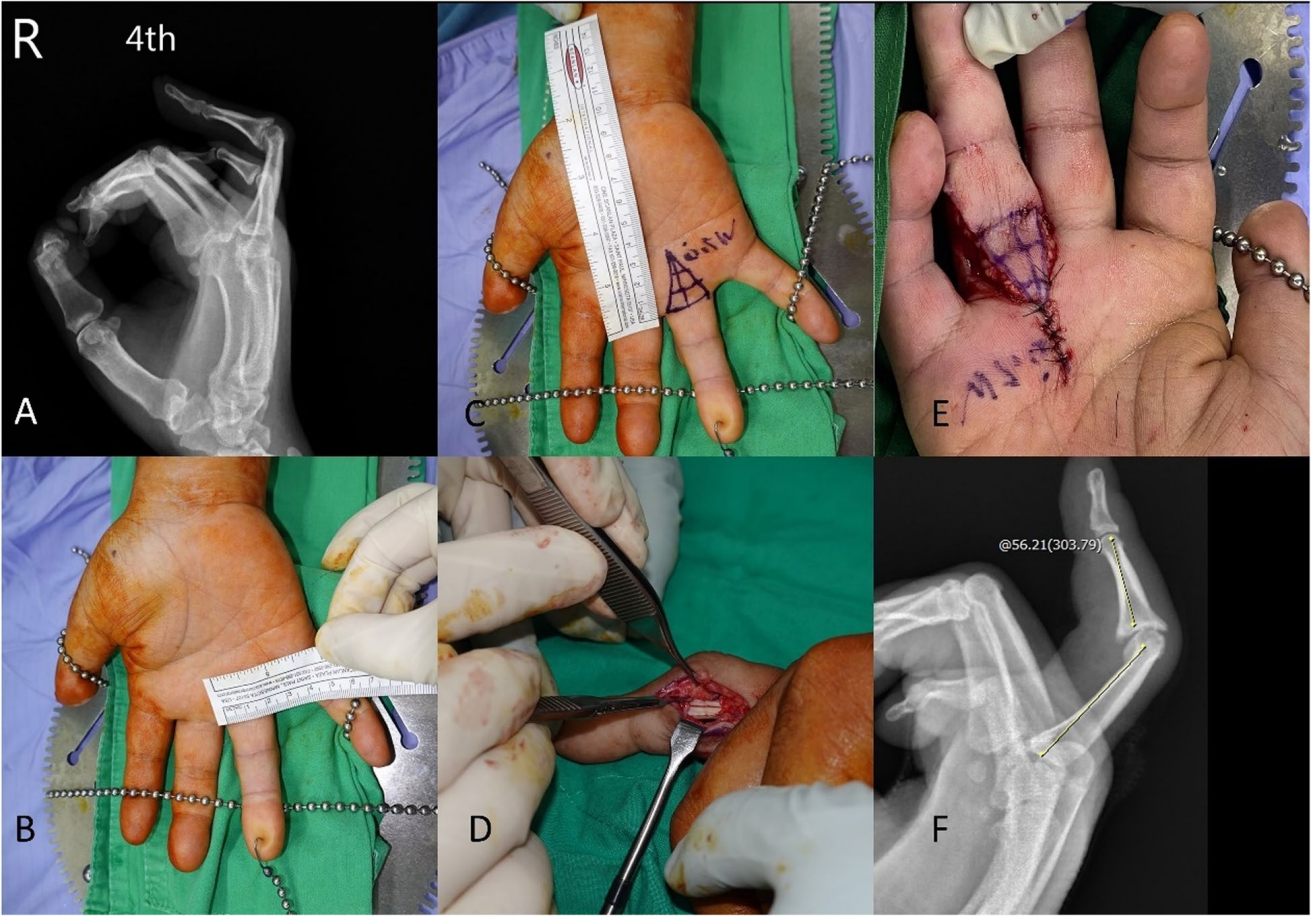
**A**. The extension lag of the patient’s PIP joint was 83°. **B**. The width of the digit base (W) was 20.0 mm. **C**. The height of the V–Y flap is 1.5 W from the base of the contracted digit, which is 30.0 mm. **D**. Tenoarthrolysis performed through bilateral mid-lateral approach. **E**. Full passive extension of the contracted digit, recorded during the surgery. **F**. Postoperative plain film showing that the extension lag of right fourth PIP joint has decreased to 56° actively

A 40-year-old man presented to our clinic with a fourth PIP joint flexion contracture on his right hand. Based on the history, the patient sustained the injury during a basketball game over 20 years ago, and post-traumatic PIP joint stiffness had bothered him since then. No fracture nor open wound was noted according to the patient’s statement. The extension lag of the joint was 83° (Fig. 5A).

In this case, we performed tenoarthrolysis and a V–Y advancement flap based on the digital artery to resolve this contracture (Figs. 5B–F). The length of M, P, H, and W was 10 mm, 20 mm, 33 mm, and 22 mm, respectively. The available advance distance of V–Y advancement flap for a 90°-contracture correction was calculated as L' $$-$$ L = $${\overline{P}}^{\prime}$$ + $$\overline{M }$$$$- \sqrt{{\overline{P}}^{{\prime}{2}}+{\overline{M} }^{2}}$$= 9.1 mm. The V–Y flap tip (α) angle was 36.9°. The actual advance distance was 9 mm and the α angle was 36°, which were very close to our prediction.

#### Patient 2

**Fig. 6:**
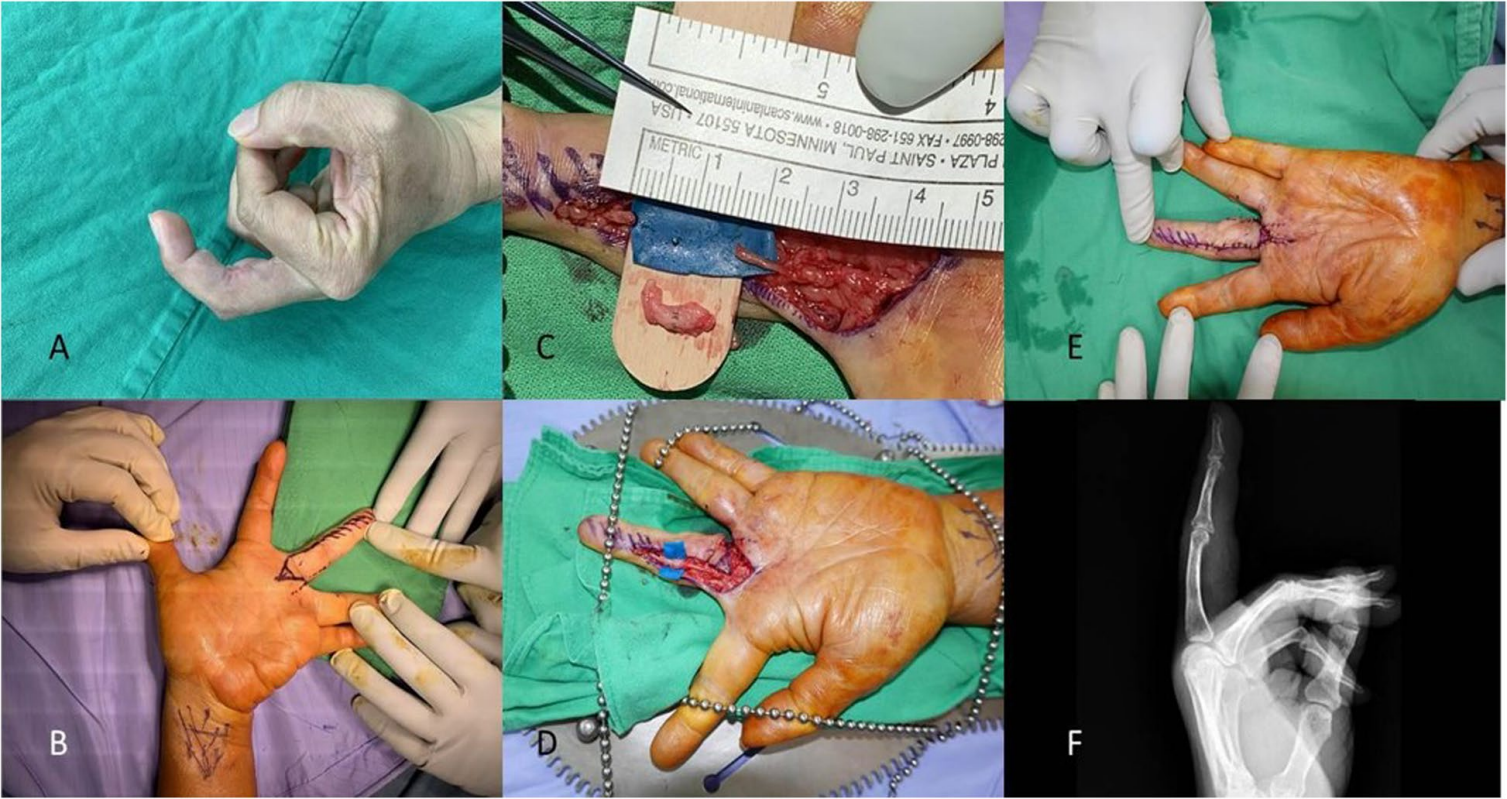
**A**. Flexion contracture of the left third PIP joint is noted with a 45° extension lag. **B**. The tip of the advanced V–Y flap is designed 27 mm proximal to the digital base. **C**. Excision of neuroma is performed. **D**. Nerve repair is done with sural nerve graft harvested from the right leg. **F**. Passive full extension of the PIP joint is noted intraoperatively. **E**. Postoperative plain film showing nearly full active extension of the left third PIP joint

A 53-year-old woman presented with post-traumatic left third PIP joint flexion contracture and joint pain of a year’s duration after undergoing flexor digitorum superficialis (FDS) and flexor digitorum profundus (FDP) tendon repair due to an accidental cutting injury. Preoperatively, the extension lag of the PIP joint was 45° (Fig. 6A), and magnetic resonance imaging (MRI) revealed a 1.5-cm neuroma of the radial digital nerve of the left third digit.

**Fig. 7:**
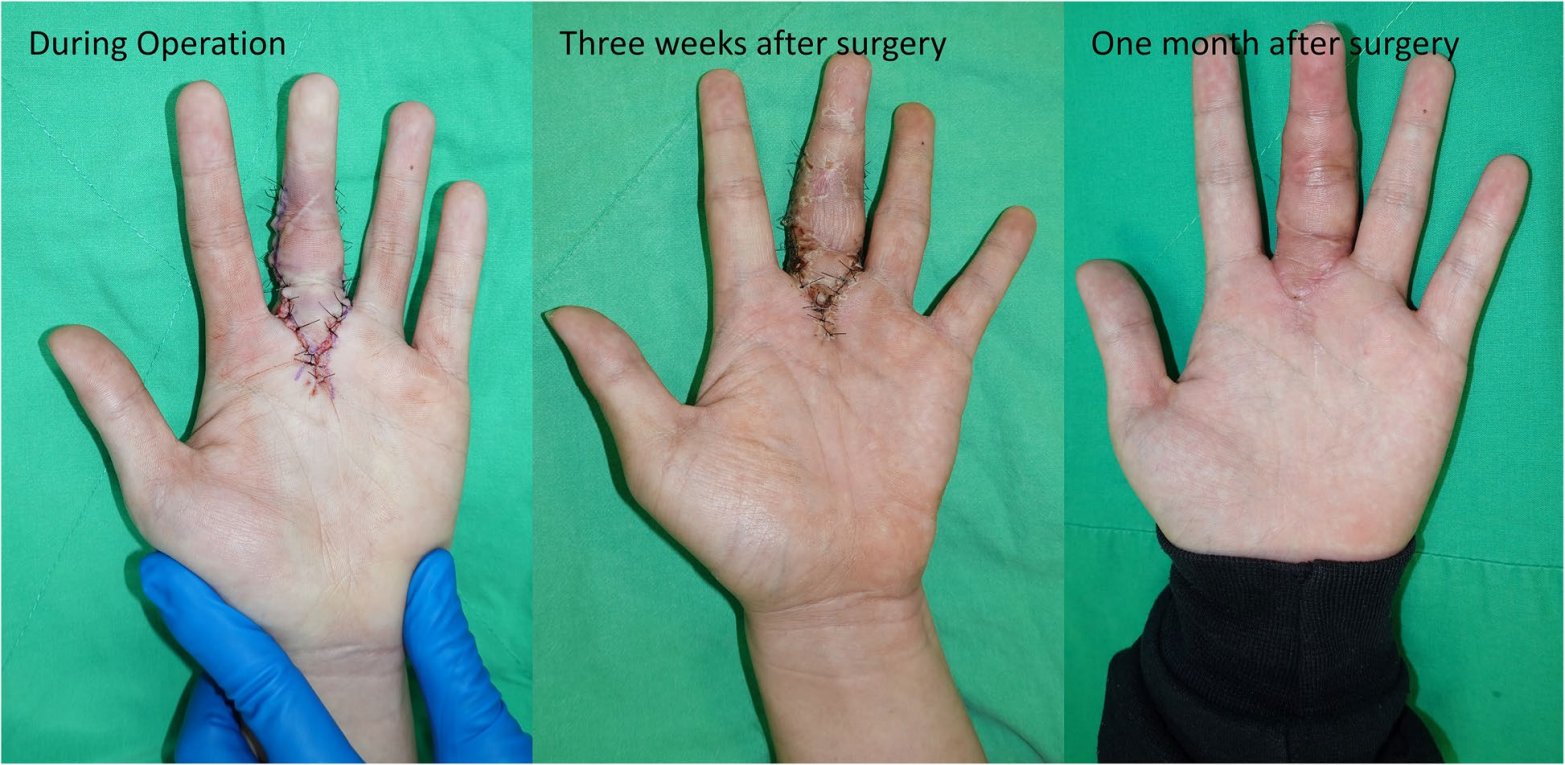
The wound healed adequately without complications postoperatively. The tension of the wound closure is acceptable without ischemic change in the flap tip

The length of M, P, H, and W was 11.5 mm, 23 mm, 30 mm, and 18 mm, respectively (Fig. 6B). The available advance distance of V–Y advancement flap for a 90°-contracture correction was calculated as L' $$-$$ L = $${\overline{P}}^{\prime}$$ + $$\overline{M }$$$$- \sqrt{{\overline{P}}^{{\prime}{2}}+{\overline{M} }^{2}}$$= 10.3 mm. The V–Y flap tip (α) angle was 36.9°. First, the patient underwent tenolysis, neuroma excision (Fig. 6C), and microscopic nerve repair with sural nerve autograft (20 mm) (Fig. 6D). Subsequently, a V–Y advancement flap was used for wound closure. No limitation in PROM was noted during the surgery, and the extension lag of the PIP joint had decreased to 0° actively (Figs. 6E and F). The actual advance distance was 8.7 mm which was not greater than the available advance distance of our method. The α angle was 36°, which were very close to our prediction. The patient subjectively claimed better function, and none of them had skin ischaemia or infection postoperatively. (Fig. 7).

As shown in Table 4. At the 3-month follow-up visit, patient 1 showed improvement in ROM of the right 4th PIP joint but still had an extension lag of 56°, which may have resulted from the laxity of the extensor tendon. Patient 2 also showed significant improvement in ROM of the left 3rd PIP joint both actively and passively; the patient had an extension lag of 45° preoperatively.

**Table 4:** Comparison of extension lag of the affected PIP joint before and 3 months after surgery

Extension lag	patient 1	patient 2
Pre-operative (°)	83.15	45.00
Post-operative (°)	56.21	13.28
AROM gained (°)	26.94	31.72

After the surgery, rehabilitation protocol was launched for achieving better AROM.

### Postoperative rehabilitation protocol

A finger splint was applied immediately postoperatively, and passive movement of PIPJ was encouraged 1 week after surgery. Stitches were removed 2 weeks after surgery, and patient began to active move finger in a limited range under the guidance of a well-trained physical therapist. At 4 weeks after surgery, full range of active movement was allowed.

## Discussion

In the geometric analysis of finger, we found that differences of finger segments exist between sexes and fingers. Male patients usually have longer finger segments; thus, longer advance distance is needed for an equal degree of flexion contracture than that of female patients. Similar findings were observed between different fingers; the middle fingers usually need more advance distance for the same degree of flexion contracture than other fingers. In our procedure, the V–Y flap including the whole length from the finger base to the distal palmar crease (H) was raised. According to our geometric model, this length was sufficient for the advance distance to correct PIP flexion contracture up to 90°. Because the advance distance was less than the H in all fingers, the tip of V would not across the palmar digital crease, which avoids the formation of contracted scar. Our procedure provides a simple, safe, and effective method for designing the digital artery-based V–Y advancement flap.

Kinoshita et al. proposed the V–Y advancement flap based on a digital artery for closure of soft tissue defects after surgical release for PIP joint flexion contracture [4]. The advance distance of the V–Y advancement flap was reported to be 25 mm for the thumb and 18 mm for the fingers to cover the skin defect after correction of the flexion contracture of the digits. However, in a recent study, Tseng et al., suggested that the advance distance should be limited to 10 to 14 mm, to avoid arterial insufficiency and nerve injury, which was much less than that proposed by Kinoshita [6]. According to our geometric model, longer fingers usually need more advance distance to correct the same degree of flexion contractures than shorter fingers. The data of the operative finger was not recorded in previous studies. Our model and finger geometric data can be used to estimate the advance distance prior to the surgery and prevent misunderstanding of “tolerable advance distance” due to individual differences. Literature review showed neurovascular bundle can be over stretched when the flap distal advancement distance longer than 10–14 mm. Therefore, based on our mathematic model, if evaluation before the operation revealed the V–Y flap not working well, more surgical procedures (skin graft, regional or distal flap) may also be taken into consideration to cure the PIP joint flexion contracture.

Traditionally, tenoarthrolysis is usually performed under general anaesthesia. The adequacy of the surgical release can only be evaluated by passive motion, which may overestimate the patient’s extension strength and result in inadequate release. Under the WALANT technique, real-time intraoperative information of passive and active ROM can be provided to the surgeons, enabling them to evaluate the necessity of further soft tissue release procedures. In patients with residual extension lag after a V–Y advancement flap based on the digital artery, reinforcement of the extensor tendon can be considered as a second surgery.

The ratio of height to base of the distal palm part of the digital artery-based V–Y advancement flap has seldom been discussed in previous studies [4, 6, 10].However, the length-to-width ratio of any flap should not exceed 2:1 to prevent blood circulation disorders or necrosis at the distal end of the flap [7]. In our study, the ring and index fingers had the largest and smallest length-to-width ratio. However, all the flap length-to-width ratio in our participants did not exceed 2:1.

There are several limitations in our study. First, our study was conducted in healthy digits and clinically practiced in a limited number of pathological digits due to the difficulty in collecting large series of pathological digits data. Second, the study was conducted without a comparison with other techniques (for example: Z-plasty). Third, the bio-properties of scarred skin were not considered in this study, and finally the extension lag may recur during the period of scar formation. Though we had put the formula put into practice, it has only been half a year since the first patient received this kind of flap surgery developed through a mathematical formula. Further mid-to-long term follow up on functional outcomes and ROM of the affected digit should be conducted and assessed.

## Conclusions

This article provides a mathematical basis and simplified method for the design of V–Y advancement flaps based on digital arteries. Based on the geometric data obtained from 120 fingers, this method provides a proper flap length-to-width ratio without skin complications in all fingers excepts the thumb. Under controlled distal advance distance of the flap according to the length of the proximal phalanx, the flexion contracture < 90° can be corrected without neurovascular complications by this method.

## Supplementary Information

**Additional file 1.**

## Data Availability

All data generated or analysed during this study are included in this published article and its supplementary information files.
